# Comparative transcriptome profiling and molecular marker development for oil palm fruit color

**DOI:** 10.1038/s41598-022-19890-2

**Published:** 2022-09-15

**Authors:** Potjamarn Suraninpong, Sunya Nuanlaong

**Affiliations:** 1 School of Agricultural Technology and Food Industry, Walailak University, Nakhon Si Thammarat, 80160 Thailand; 2 Biomass and Oil Palm Center of Excellence, Walailak University, Nakhon Si Thammarat, 80160 Thailand

**Keywords:** Biotechnology, Plant sciences

## Abstract

Oil palm harvesting is normally determined by fruit exocarp color. To detect expressed sequence tag (EST)-simple sequence repeat (SSR) markers in oil palm hybrid populations, de novo transcriptomic profiling of Nigeria black and Suratthani 1 (Deli × Calabar) plants was performed. More than 46 million high-quality clean reads with a mean length of 1117 bp were generated. Functional annotation and gene ontology (GO) enrichment analysis of differentially expressed genes (DEGs) revealed that the genes were involved in fruit color development and pigment synthesis. Comparison of immature/mature DEGs indicated that nigrescent fruit color was driven by the anthocyanin biosynthesis pathway (ABP); however, the carotenoid biosynthesis pathway (CBP) was involved in the color development of both fruit types. The transcripts of both unique and different genes involved in the ABP and CBP in higher plants were highlighted for further study, especially 3GT, downstream genes in the ABP, and DEARF27 in the CBP. Additionally, SSR primer motifs, namely, 9949, discovered from the DEGs upregulated in the virescent type that encode vacuolar iron transporter (VIT), could separate the nigrescence and virescence traits of Nigeria hybrids. This novel primer has potential to be used as a molecular for further selection in breeding programs especially involving the specific genetic backgrounds described in this study.

## Introduction

Oil palm (*Elaeis guineensis*) trees produce edible vegetable oil derived from the mesocarp (reddish pulp) of the fruit. Palm oil is rich in carotenoids, from which it derives its deep red color, and the major component of its glycerides is the saturated fatty acid palmitic acid. The harvesting and transportation operations of palm oil are of great importance for palm oil production. Harvesting of oil palm normally occurs when approximately 10 fruits per bunch drop, which is a sign of their maturity. In practice, the change in oil palm fruit exocarp color is used to determine the harvesting stage^1^. In general, the nigrescent type is the most commonly planted type, followed by the virescent type, whereas the albescent type is the least commonly planted. Unripe nigrescent fruit have a black–brown color toward the top and an ivory color at the base; when ripe, the fruit color turns deep reddish orange at the top, but there is no change color at the base. Unripe virescent fruit are green, but when ripe, they have a light reddish orange color^2^. The nonsignificant difference in the color of nigrescent fruit from the unripe to ripe stages leads to inaccurate predictions of harvest time. Thus, oil palm breeders are interested in virescent fruit, whose maturity is readily apparent. A gene that has been reported to be involved in the anthocyanin biosynthetic pathway (ABP) of oil palm fruit exocarp color is virescens (*VIR*)^3^. These findings inspired us to compare genes that are differentially expressed between nigrescent types and virescent types at the unripe and ripe stages to develop molecular markers for virescent types among our hybrid population generated from a cross between Nigeria black (a nigrescent type) and Suratthani 1 (Deli × Calabar, a virescent type).

DNA sequencing is fundamental to research on the structures and functions of cells. This technique could help biologists in a broad range of applications, such as molecular cloning, breeding, identifying pathogenesis-related genes, and performing comparative and evolutionary studies^4^. In oil palm, utilization of transcriptome information for many purposes has been reported^5–11^. Next-generation sequencing (NGS), particularly de novo transcriptome sequencing, provides access to large amounts of genomic data, with a relatively high rate of transferability for non-sequenced genomes, and is an effective tool for the development of molecular markers, especially simple sequence repeats (SSRs). SSRs derived from expressed sequence tag (EST)-SSRs are highly polymorphic, reproducible and reliable and undergo codominant inheritance; therefore, SSRs are among the most suitable markers for detecting genetic diversity, determining phylogenetic relationships, mapping the genome, constructing linkage maps and analyzing population genetics^12,13^.

In this study, we constructed a reference transcriptome of immature/mature nigrescent and virescent morphotypes, compared differentially expressed genes (DEGs) involved in the ABP and the carotenoid biosynthesis pathway (CBP), and used EST-SSR markers to provide support for oil palm breeders in the selection of a Nigeria black hybrid population. Our findings concerning ABP and CBP provide initial data, but further insights into the molecular and functional biology of the genes involved in both pathways are needed for application in higher plants. In addition, the obtained markers have potential to be applied in our palm hybrid population.

## Results

### Transcriptome sequencing and de novo assembly

High-throughput sequencing was used, resulting in the generation of 48,017,160–66,162,228 pairs and 46,896,912–64,521,874 pairs of raw reads and clean reads from the nigrescent-type library and the virescent-type library, respectively, with amounts of clean bases ranging from 7.0 to 9.7 Gb, respectively. Quality control analysis of the data revealed a minimum error base rate of 0.01% with an average quality value and Q20 and Q30 scores greater than 97% and 93%, respectively. The CG content of the clean reads ranged from 48.61 to 50.16% (Suppl. 1). Based on the high-quality clean reads, a total of 143,797 transcripts and 143,668 unigenes were identified. Of these, 46,664 unigenes with a size between 500 and 1000 bp were the most common. A total of 160,423,261 nucleotides were assembled, which had a mean length of 1117 bp, an N50 of 1664 bp and an N90 of 505 bp (Table 1).

**Table 1:** Statistics of the assembly quality.

Type	Transcripts	Unigenes
Total number	143,797	143,668
Total number of sequence bases	160,456,634	160,423,261
200–500 bp	43,740	43,614
500–1000 bp	46,667	46,664
1–2 kbp	32,113	32,113
> 2 kbp	21,277	21,277
Minimum length	201	201
Mean length	1116	1117
Medium length	734	734
Maximum length	19,990	19,990
N50	1664	1664
N90	505	505
Total nucleotides	160,456,634	160,423,261

### Functional annotations of unigenes

Among the 143,668 unigenes, 95.048 unigenes (66.15%) were the most significant in the NCBI nucleotide sequence (Nt) database, followed by the NCBI nonredundant protein (NR) (76,996 unigenes, 53.59%), SwissProt (62,143 unigenes, 43.25%), gene ontology (GO) (58,842 unigenes, 40.95%), Protein family (Pfam) (58,677 unigenes, 40.85%), Kyoto Encyclopedia of Genes and Genomes (KEGG) Ortholog (KO) (31,191 unigenes, 21.71%) and euKaryotic Ortholog Groups (KOG) (29,939 unigenes, 20.83%) databases (Table 2). A total of 17,374 unigenes (12.09%) matched entries in five of the seven databases (Suppl. 2). BLASTX similarity analysis of the 143,668 unigenes revealed 33.4% (47,985 unigenes) significant homology (E-values < 1e^−100^) with the other sequences in the NR database, with the highest similarity distribution of unigenes ranging from 80 to 90%. The NR annotation species distribution analysis revealed homology with sequences of *E. guineensis* (63.9%), *Phoenix dactylifera* (9.2%) and *Pyrus* × (7.0%) (Suppl. 3).

**Table 2:** Summary of the annotation information of unigenes related to oil palm fruit exocarp color in public databases.

Sequence database	Number of annotated unigenes	Percentage of annotated unigenes
NR	76,996	53.59
NT	95,048	66.15
KO	31,191	21.71
SwissProt	62,143	43.25
Pfam	58,677	40.84
GO	58,842	40.95
KOG	29,939	20.83
All	17,374	12.09
At least	102,546	17.37
Total unigenes	143,668	100.00

Additionally, the 143,668 unigenes were annotated into three major categories, i.e., biological processes, molecular functions and cellular components, with 57 subcategories (Suppl. 4). Most unigenes in the biological process category were specific to cellular processes (33,805 unigenes), metabolic processes (30,781 unigenes) and single-organism processes (24,691 unigenes). In the molecular function category, most of the genes were implicated in binding (33,217 unigenes), followed by catalytic activity (25,917 unigenes) and transporter activity (4021 unigenes). In the cellular component category, cells (18,594 unigenes) were the most represented, followed by cell parts (18,592 unigenes) and macromolecular complexes (12,211 unigenes).

In addition, KOG function classification annotated 29,939 unigenes into 25 categories, among which the majority of genes (4459 unigenes) were associated with general function prediction only, followed by posttranslational modification, protein turnover, chaperones (4037 unigenes) and signal transduction mechanisms (2933 unigenes) (Suppl. 5).

Next, the unigenes were mapped to the KEGG pathway database to identify the biological pathways. In total, 31,191 unigenes were classified into 21 secondary pathways of five primary pathways. Metabolism-related pathways were the majority of these pathways (10 pathways), followed by genetic information processing (4 pathways). Among these unigenes, the translation category had the largest number of unigenes (3008 unigenes), followed by the carbohydrate metabolism category (2498 unigenes). The folding, sorting and degradation category (2430 unigenes) was the smallest group (Suppl. 6).

Moreover, when the unigenes in the transcription factor group were analyzed, 4805 unigenes were ultimately mapped to 80 transcription factor families. Of these, the C2H2 transcription factor was the most abundant (390 unigenes), followed by the MYB transcription factor (298 unigenes), C3H transcription factor (232 unigenes), AP2-EREBP transcription factor (225 unigenes), and orphan transcription factors (204 unigenes) (Suppl. 7).

### Identification and functional annotation of DEGs

**Figure 1:**
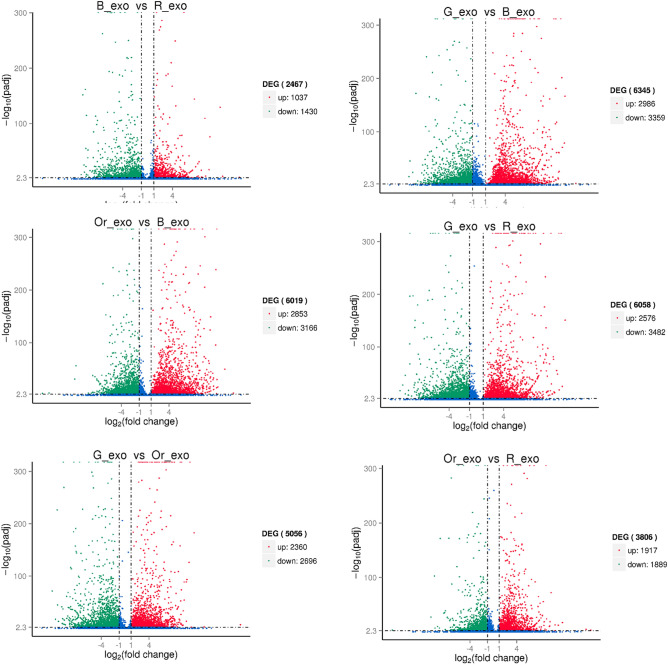
Volcano plot of nigrescent and virescent oil palm fruit in each fruit development stage. (G_exo = green immature virescent type, Or_exo = orange mature virescent type, B_exo = black immature nigrescent type and R_exo = red mature nigrescent type).

DEGs were analyzed after normalization of the read count of each unigene. The percentage of total mapped reads of each sample ranged from 75 to 80%. G_exo had the highest total mapped reads (Suppl. 8). Most of the DEGs were downregulated, and there were no correlations. The G_exo:B_exo comparison group contained the most DEGs (6345 genes), with 3359 downregulated genes. However, the O_exo:R_exo comparison group contained more upregulated genes (1917 genes) than downregulated genes (1889 genes) (Fig. 1). To determine the functions and functional patterns of the unknown genes, correlations of the DEGs were determined. The analysis results showed four different gene expression patterns according to fruit color. B_exo presented a DEG pattern that was similar to that of R_exo, while G_exo presented a DEG pattern similar to that of Or_exo (Suppl. 9).

**Figure 2:**
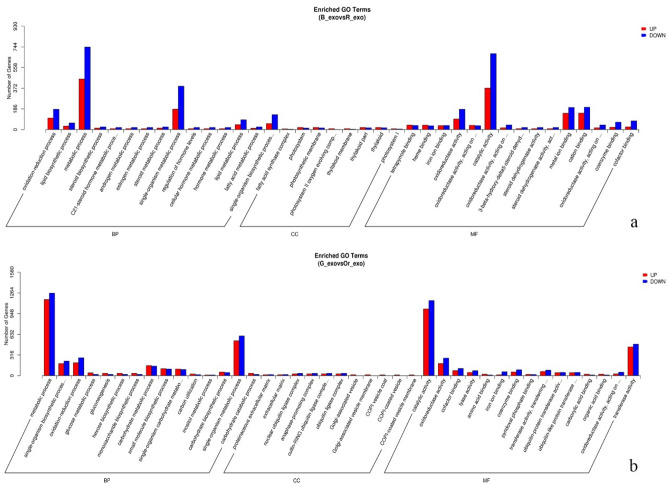
GO terms of genes differentially expressed between oil palm fruit types and fruit development stages. (**a**) Nigrescent immature/mature type. (**b**) Virescent immature/mature type. (BP = biological process, CC = cellular component, MF = molecular function).

**Figure 3:**
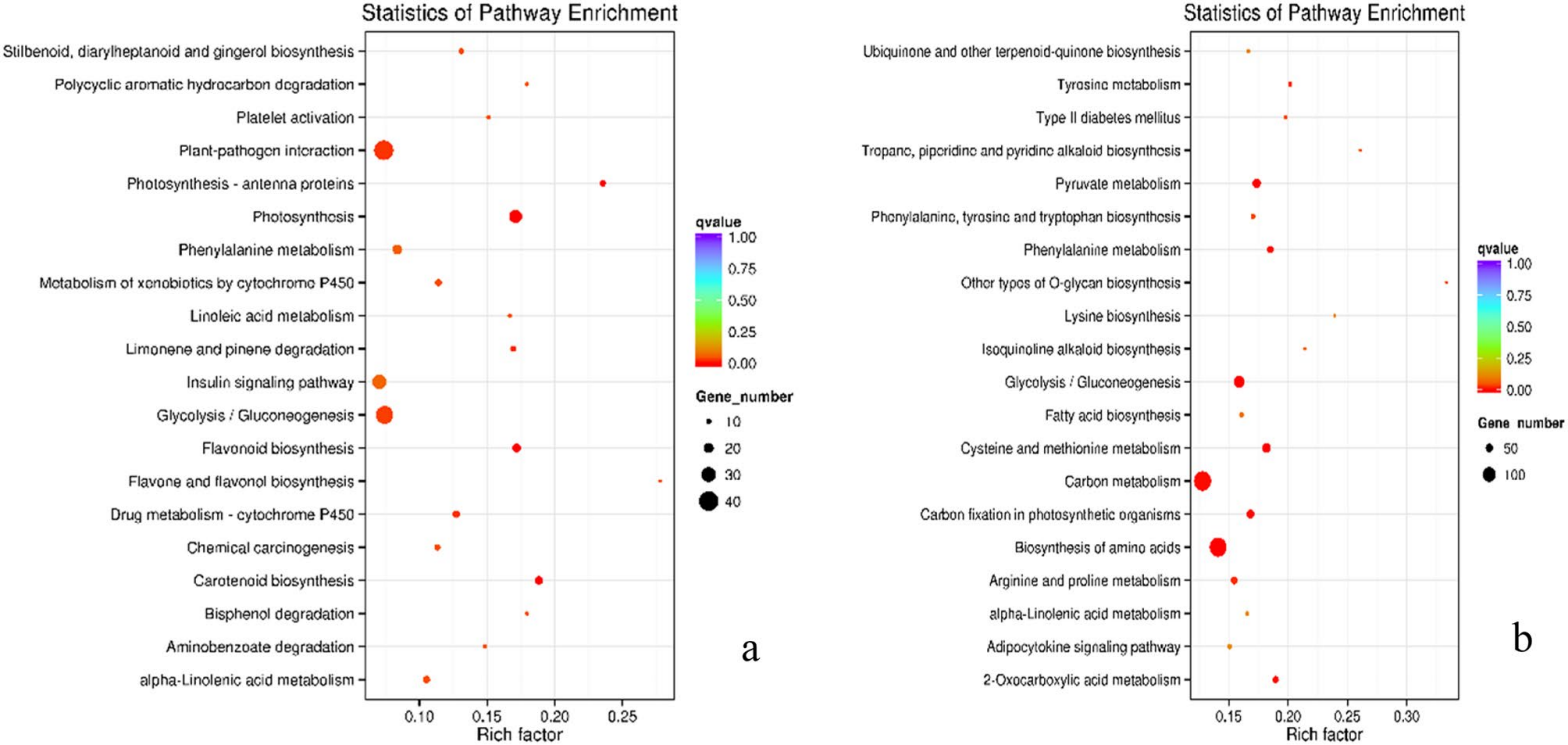
KEGG pathway enrichment analysis of genes differentially expressed between oil palm fruit types and at different fruit development stages. (**a**) Nigrescent immature/mature type. (**b**) Virescent immature/mature type.

Furthermore, GO enrichment analysis of the genes differentially expressed between each morphotype (B_exo:R_exo and G_exo:Or_exo) revealed similar results at each GO level. In the biological process category, metabolic processes were associated with the highest gene expression, followed by single-organism metabolic processes. In the molecular function category, catalytic activity was associated with the highest gene expression, followed by transferase activity (virescent type), metal ion binding (nigrescent type) and cation binding (nigrescent type) (Fig. 2). In contrast, KEGG pathway enrichment analysis of the DEGs revealed significant differences in each type. In the virescent type, the genes involved in the carbon metabolism pathway were the most highly expressed, followed by those involved in the biosynthesis of amino acids pathway. In the nigrescent type, most DEGs were associated with plant–pathogen interactions followed by glycolysis/gluconeogenesis (Fig. 3).

### Comparison of genes differentially expressed between nigrescent and virescent types

**Figure 4:**
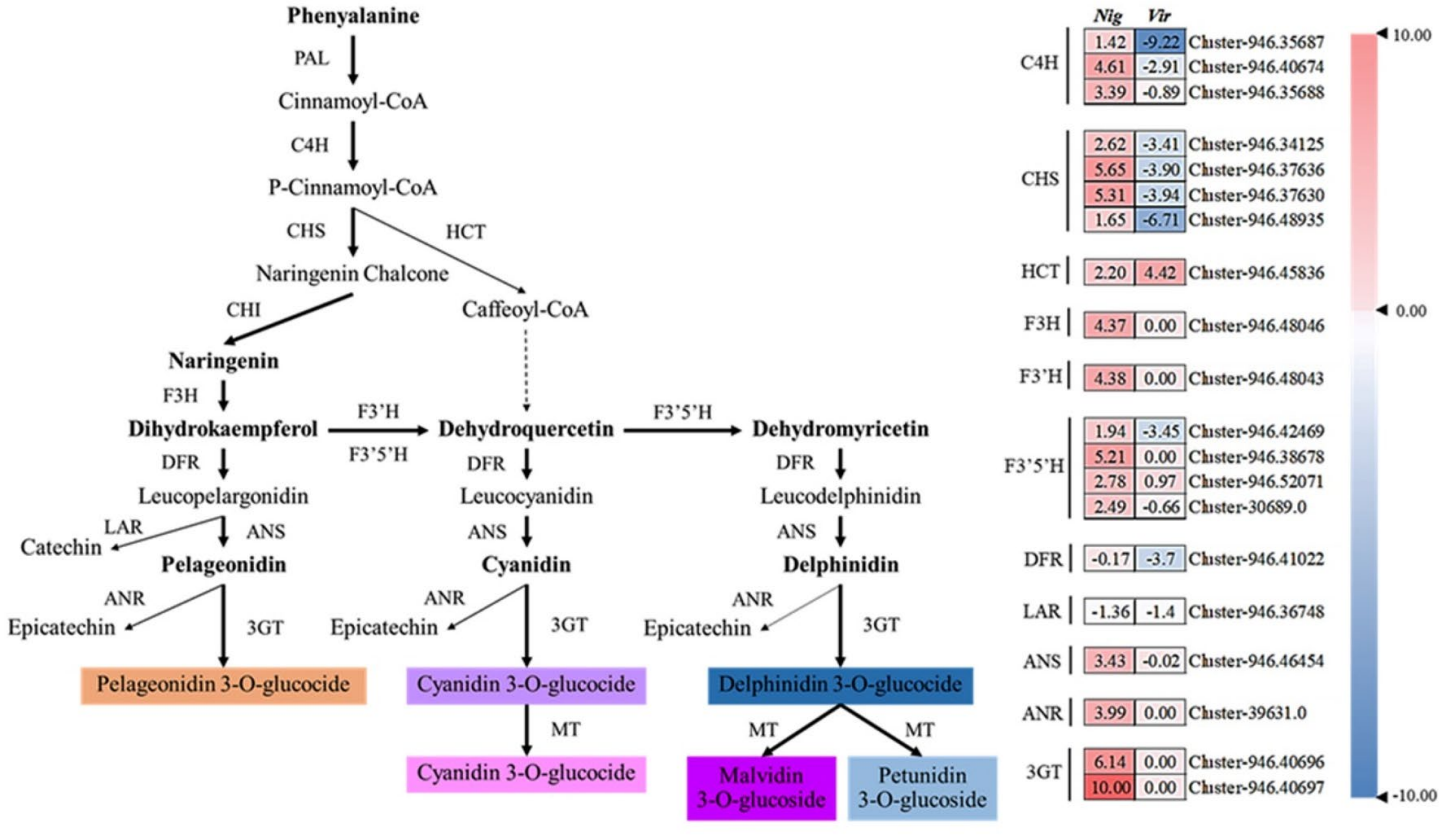
Simplified scheme and heatmap of the expression of genes related to flavonoid and anthocyanin biosynthesis in the exocarp of oil palm fruit of nigrescent (*Nig*) and virescent (*Vig*) types. Each colored cell represents the average log_2_(FPKM) value of each pathway gene according to the color scale. PAL, phenylalanine ammonia-lyase; C4H, cinnamic acid 4-hydroxylase; CHS, chalcone synthase; CHI, chalcone isomerase; HCT, shikimate O-hydroxycinnamoyl transferase; F3H, flavanone 3-hydroxylase; F3′H, flavonoid 3-monooxygenase; F3′5′H, flavonoid 3′, 5′-hydroxylase; DFR, dihydroflavonol-4-reductase; LAR, leucoanthocyanidin reductase; ANS, anthocyanidin synthase; ANR, anthocyanidin reductase; 3GT, flavonoid 3-O-glucosyltransferase; MT, methyl transferase.

**Figure 5:**
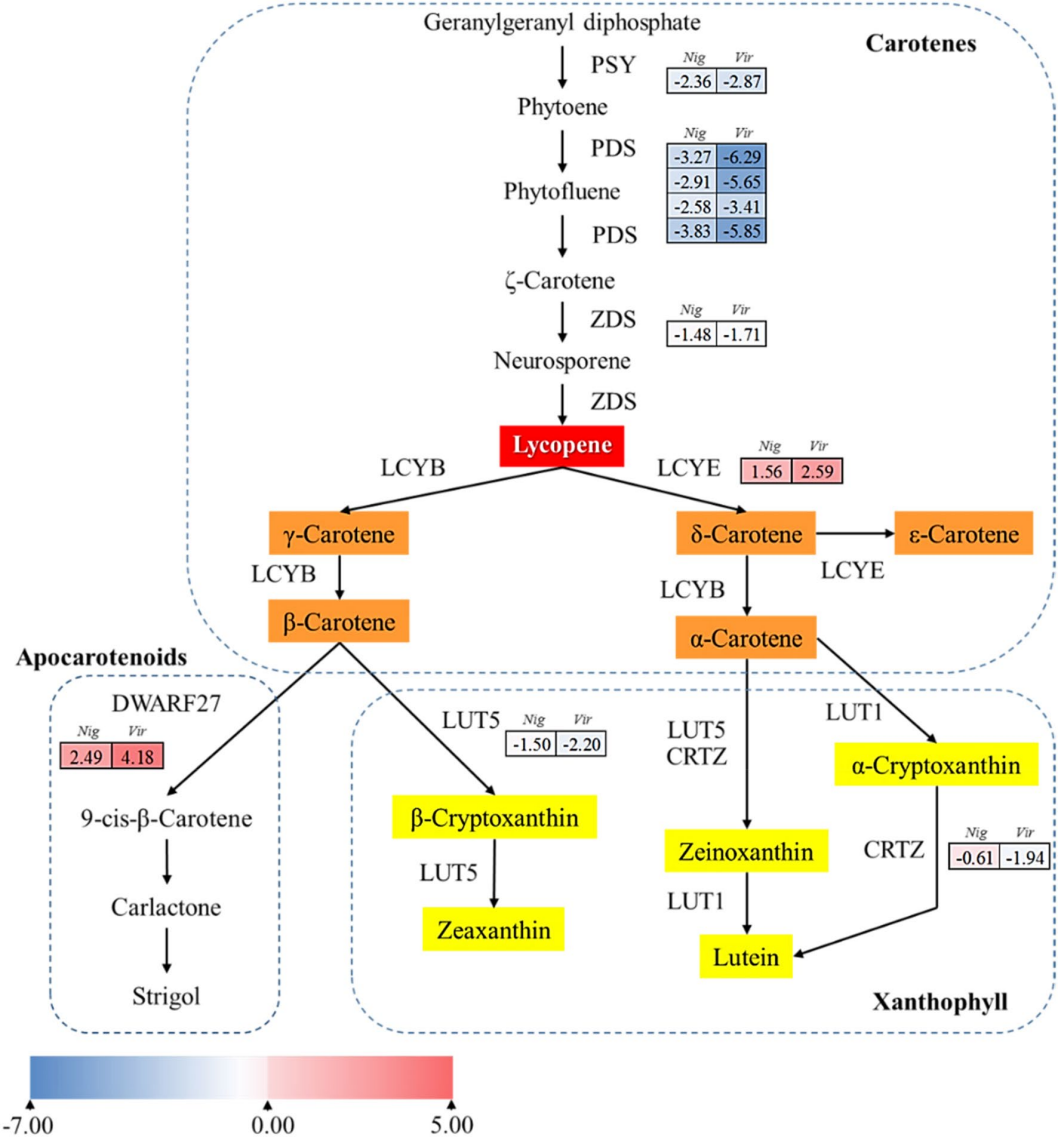
Simplified scheme and heatmap of the expression of genes related to carotenoid biosynthesis in the exocarp oil palm fruit of nigrescent (*Nig*) and virescent (*Vig*) types. Each colored cell represents the average log_2_(FPKM) value of each pathway gene according to the color scale. PSY, phytoene synthase; PDS, phytoene desaturase; ZDS, ζ-carotene desaturase; LCYE, lycopene ε-cyclase; LCYB, lycopene β-cyclase; LUT1, carotene ε-ring hydroxylase; LUT5, β-ring hydroxylase; CRTZ, β-carotene 3-hydroxylase; DWARF27, β-carotene isomerase.

Annotations of the DEGs involved in the ABP revealed higher transcription at the immature stage than at the mature stage, especially in the nigrescent type, resulting in upregulated gene expression. The genes involved in the ABP were cinnamic acid 4-hydroxylase (C4H), chalcone synthase (CHS), flavanone 3-hydroxylase (F3H), flavonoid 3-monooxygenase (F3’H), flavonoid 3′, 5′-hydroxylase (F3′5′H), dihydroflavonol-4-reductase (DFR), leucoanthocyanidin reductase (LAR), anthocyanidin synthase (ANS), anthocyanidin reductase (ANR), flavonoid 3-O-glucosyltransferase (3GT) and shikimate O-hydroxycinnamoyl transferase (HCT). The most regulated in this pathway was 3GT. In addition, both fruit types were found to contain two homologs of 3GT, three homologs of C4H, and four homologs of CHS and F3′5′H (Fig. 4). In contrast, the annotations of the DEGs involved in CBP revealed higher transcription of genes at the mature stage than at the immature stage, especially in the virescent type, resulting in the downregulation of genes such as phytoene synthase (PSY), phytoene desaturase (PDS), ζ-carotene desaturase (ZDS), β-ring hydroxylase (LUT5) and β-carotene 3-hydroxylase (CRTZ). Lycopene ε-cyclase (LCYE) and β-carotene isomerase (DWARF27) were highly upregulated in the virescent type compared with the nigrescent type (Fig. 5).

### EST-SSR discovery

**Figure 6:**
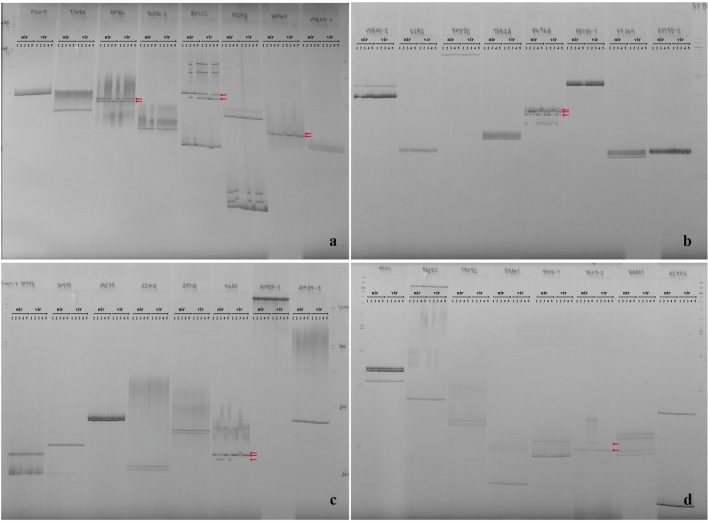
Polymorphisms of DNA bands after amplification with 6 primer pairs and separation by 5% denaturing polyacrylamide gel electrophoresis. (**a**) Primer 20,186, 54,322 and 55,957, (**b**) Primer 76,163, (**c**) Primer 9949, **(d)** Primer 8605–2. nir = nigrescent type of Nigeria Black, vir = virescent type of Deli × Calabar. 1–5 = replication in each morphotype. The red arrows indicate the different alleles.

A total of 902 genes differentially expressed between immature and mature virescent types were employed for primer design, of which 114 genes (144 positions) (12.64%) had SSR-like positions. As expected, trinucleotide repeat motifs were the most frequent type of microsatellite (55; 38.19%). The other repeat motifs occurred at much lower frequencies, i.e., there were 42 (29.17%), 35 (24.31%), 7 (4.86%) and 5 (3.47%) dinucleotide, hexanucleotide, pentanucleotide and tetranucleotide repeats, respectively. However, 82 primers (56.94%) had flanking sequences sufficient for designing appropriate unique primers (Suppl. 10). Fifty out of these 82 primer (60.98%) pairs were effectively amplified. Of these, six (12%) were polymorphic, with 2–4 alleles per SSR and ten genotype patterns (Fig. 6). Nevertheless, the allele frequency and genotype frequency evaluations revealed that only one SSR marker could distinguish between the nigrescent and virescent types. Marker 9949 revealed alleles A, B and C. In the nigrescent type, alleles B and C had the highest BC genotype (0.6). However, in the virescent type, alleles A and B were found, and the highest BB genotype was 0.8. (Suppl. 11).

## Discussion

Fruit ripening is an irreversible and continuous process involving physiological and biochemical changes that ultimately result in the development of soft, high-quality fruits. In oil palm, fruit maturation is very important, as there is a high accumulation of fatty acids at the harvest stage. RNA sequencing (RNA-seq) technology was applied in this study to identify the key roles of metabolic pathways between immature and mature plants and to explore the transcriptomic differences between two contrasting cultivated oil palm genotypes, after which EST-SSRs for oil palm fruit exocarp color were developed. We constructed four RNA libraries that presented a low error rate base of 0.01%, resulting in a high quality of unigenes compared with those of previous studies^14,15^. Thus, a more powerful information database with a higher capacity for the analysis of expressed genes, especially for those involved in the ABP and CBP, and for SNPs was generated. Furthermore, the annotation results from all the databases together with the significant homologs of unigenes from *E. guineensis* (63.9%) confirmed the reference genome sequence for further analysis.

The GO enrichment analysis of our four libraries revealed high representation in biological process, molecular function, and cellular component categories, indicating that fruit development and fruit exocarp color are associated with energy and biosynthesis activity together with metabolic processes in a wide variety of organelles. Additionally, searches of our sequence data against the sequence information in the KOG database for classification of groups of proteins that could be orthologous or paralogous to the sequences in the eukaryotic protein database and against the information in the KEGG database to characterize the active biological pathways revealed the same proteins and biological mechanisms. These predicted pathways are responsible for fruit development and pigment accumulation via compound biosynthesis, degradation, utilization and assimilation.

In addition, genes involved in the ABP and CBP were found to not only be structural genes but also regulatory genes. Transcription factors are considered critical in the regulation of anthocyanin biosynthesis- or carotenoid biosynthesis-related structural genes and their accumulation. Among DEGs identified in this study that encode transcription factors, C2H2 was the most abundant, followed by MYBs, and those encoding bHLH transcription factors were the seventh most abundant. C2H2 is the transcription factor responsible for the mechanism underlying the coloration associated with fruit ripening. However, most MYB, basic helix-loop-helix (bHLH) and WD repeat (WDR) transcription factors have been reported to regulate anthocyanin biosynthesis and accumulation^16,17^. The identification of MYB as one of the DEG was in agreement with a previous report by Singh et al.^3^ that found that the *VIR* gene controls fruit color and encodes an R2R3-MYB in oil palm. *VIR*, an R2R3-MYB transcription factor, is homologous to LhMYB12 in lilium (*Lilium candidum*) and PAP1 and AtMYB113 in Arabidopsis (*Arabidopsis thaliana*), and was reported to control the accumulation of anthocyanins by regulating biosynthetic genes^18–20^. Thus, our results confirmed that MYB transcription factors play a critical role in the regulation of anthocyanin biosynthesis and accumulation in oil palm fruit exocarp color.

In terms of basic molecular information about the ABP in this study, the regulation of genes involved in this pathway (Fig. 4), especially in the nigrescent fruit type, is similar to the anthocyanin phenylpropanoid pathway that was previously reported^3^. The annotation of eight weeks after anthesis (WAA) of oil palm fruit color found VIR, 4Cl, CHI and leucoanthocyanidin dioxygenase 1 (*LDOX*), which did not appear in our study at four and fifteen weeks after fertilization. The differences observed was likely due to the different time points sampled during fruit development and also dissimilar genetic backgrounds used in both studies to identify gene(s) regulating oil palm exocarp color. Interestingly, the upregulation together with the redundancy of C4H, CHS, F3H, F3′H, F3′5′H, ANS, ANR and 3GT in oil palm fruit color development, as observed in our present study confirmed that not only anthocyanin biosynthesis but also polyphenol biosynthesis was involved in determining oil palm mesocarp fruit color at the ripening stage^3^. Notably, all of these genes are highly expressed in the pathway, especially 3GT. The data obtained in this study is an excellent source to develop molecular markers for predicting fruit color. However, validation of the expression observed for some of the genes via RT-PCR would add confidence in developing appropriate markers linked to these genes for use in breeding.

Furthermore, the CBP of oil palm fruit color, which is shown in Fig. 5, is similar to that previously reported^21^ but differed slightly in certain transcribed genes and in the gene expression levels observed. The divergence of gene transcripts is likely due to the different stages of mesocarp fruit color development. A previous study^21^ reported that total mesocarp carotenoid could be detected after 120 days after pollination (DAP) and reached highly amounts at 160 DAP. Thus, at that stage, the upregulation of all upstream, PSY, PDS, ZDS and phytoene desaturation (PTOX), and downstream genes, LCYE, LCYB and CYP97A, were highly evident. Compared with our study at 15 weeks and/or 105 days, all of genes in the upstream and the downstream of the pathway seem to be downregulated in virescent compared to nigrescent, excepted for DWARF27 that was highly regulated in virescent compared to nigrescent. The observed results are contributed by diverse genes expressed at different time points during fruit development. In addition, horticultural crop species normally contain 2–3 *PSY* genes that exhibit tissue-specific expression^22^ in the CBP pathway, but in oil palm, only one *PSY* gene was present. In addition, the most interesting finding is that there are four homologs of the *PDS* gene involved in the oil palm CBP that have not been previously reported in horticultural crop species or in a previous study^21^. In general, α-carotene is sequentially catalyzed primarily by CYP97-type hydroxylases or nonheme di-iron β-carotene hydroxylase (HYD) via zeinoxanthin to produce lutein, and β-carotene is hydroxylated by CHYB to produce zeaxanthin^23,24^. In this study, the production of zeaxanthin was occurred by the hydroxylase of β-carotene to zeaxanthin via β-cryptoxanthin by LUT5, not CHYB. Notably, lutein was synthesized via two processes. First, LUT5 together with CRTZ converted α-carotene to zeaxanthin and, subsequently, to lutein by LUT1. Second, LUT1 converted α-carotene to α-cryptoxanthin and, subsequently, to lutein by CRTZ. Previous research reported that there are two types of hydroxylases, CHYB (BCH) and CYP97 (CYP97A and CYP97C), which have activity on compounds with β- and ε-rings, respectively. The enzyme CRTZ belongs to the CHYB type, whereas LUT1 and LUT5 belong to the CYP97 type. LUT5 has higher biological activity on the β-ring of α-carotene but lower catalytic activity on the β-ring of β-carotene. LUT5 is more sensitive to strong light than is LUT1^25^. Thus, LUT5 might play a synergistic role in promoting zeaxanthin and lutein formation in oil palm. The downregulation of upstream genes (PSY, PDS and ZDS), the upregulation of downstream genes (LCYE) and a lack of regulation of LCYB in both nigrescent and virescent types to produce xanthophyll in this study confirmed the activity without requiring that all biosynthetic steps display high and regulated transcript abundance in the oil palm CBP pathway^21^.

In general, oil palm fruit is a major source of carotenoids (500–700 ppm), and the mesocarp of fruit from *E. guineensis* (a tenera type) comprises 1.27% phytoene, 0.06% phytofluene, 0.69% *ζ*-carotene, 0.29% neurosporene, 1.30% lycopene, 0.33% γ-carotene, 0.83% δ-carotene, 56.02% β-carotene and 35.16% α-carotene^26,27^. Lutein is the major carotene at 7 days after anthesis (DAA), with only trace amounts of α- and β-carotene, but α- and β-carotene were detected at higher levels after 21—126 DAA^28^. However, at 126 DAA and below, unripe fruit, were not detected other carotenes, i.e., phytoene, lycopene, α-zeacarotene, β-zeacarotene, neurosporene, δ-carotene, γ-carotene, ξ-carotene or phytofluene in the crude palm oil (CPO)^28^. Ripe oil palm fruit at 140 DAF presented sharply decreased lutein contents, whereas the contents of α- and β-carotene and other carotenes increased sharply^28^. Thus, it could be concluded that the development of oil palm fruit exocarp color via CBP occurred at the mature stage and not at the immature stage of either morphotype.

Interestingly, high transcript levels of DEARF27 were found in the exocarp of the oil palm fruit of the virescent type compared with the nigrescent type. DEARF27 is an isomerase that catalyzes the reversible interconversion of all-*trans*-β-carotene to 9-*cis*-β-carotene to the strigolactone (SL) biosynthesis intermediate carlactone^29^. SLs are a relatively new class of plant hormones that determine shoot branching, regulate developmental processes, establish root system architecture and promote senescence, and they are involved in pathogen defense and the abiotic stress response^30^. The current findings are the first in oil palm; thus, further investigation of the mechanism and the response of DEARF27 is needed for further application.

To date, the development of SSR markers generated via ESTs through sequencing technology has been applied in various plant species. In the current study, EST-SSRs were evaluated for their usefulness in determining genetic relationships between two morphotypes of oil palm fruit. In this study, only one primer, primer 9949, indicated the possibility of differentiating virescent and nigrescent morphotypes with clear identification of allele frequency and genotype frequency. The BLASTX of primer 9949 showed similarity with vacuolar iron transporter 1.1 [*Elaeis guineensis*], sequence ID: XP_010905459.1. Vacuolar iron transporters are involved in the transfer of iron ions from the cytosol to the vacuole for intracellular iron storage. In Arabidopsis vacuolar iron transporter 1 (*VIT*1) is highly expressed in ripening seeds^31^. In contrast, *TgVIT*1 in tulip (*Tulipa gesneriana*)^32^, *TgVIT*1 in *Cyclamen persicum* and *C. purpurascens*^33,34^ and *CcVIT* in cornflower (*Centaurea cyanus*)^35^ are responsible for blue coloration in petal cells through iron accumulation. *TgVIT*1 facilitates the coexistence of iron ions, delphinidin 3-rutinoside and delphinidin 3,5-diglucoside in petals^33,34^. Normally, flowers containing delphinidin-type anthocyanidins include delphinidin, petunidin and malvidin derivatives in which the 3′ and 5′ positions of the B-ring are hydroxylated and/or methoxylated and do not always produce a blue color. The color is instead often purple, mauve, or pink in such flowers; control of elevation of vacuolar pH and/or coexistence with metal ions is considered effective for blue coloration. Under relatively low pH conditions, pH 4–5 is suitable for blue color development due to the interaction of delphinidin 3-glucoside, as shown by Yoshida et al.^18^. In this study, five samples each of the different exocarp color were examined. However, additional samples, including from different genetic backgrounds, may need to be tested to further validate the marker identified.

## Conclusion

We generated a de novo-assembled transcriptome of nigrescent and virescent oil palm fruit morphotypes whose color starkly contrasted. Our explored of the DEG that associated with the ABP sharply indicated fruit color development of the nigrescent type, while the DEG finding from the CBP illustrated genes involved in fruit color development in both types. Notable, the highly expression of 3GT and DWARF27, the downstream genes involved in ABP and CBP, respectively, was the new highlighted data in higher plant that functional studies need to examine further. In addition, data obtained from SSR technology based on NGS is an excellent source for molecular markers development predicting fruit color. Hence, our findings provide important clues not only for understanding the molecular mechanisms underlying oil palm fruit exocarp color but also for improving the speed and accuracy of oil palm breeding programs.

## Materials and methods

### Plant material, RNA extraction and library construction

**Figure 7:**
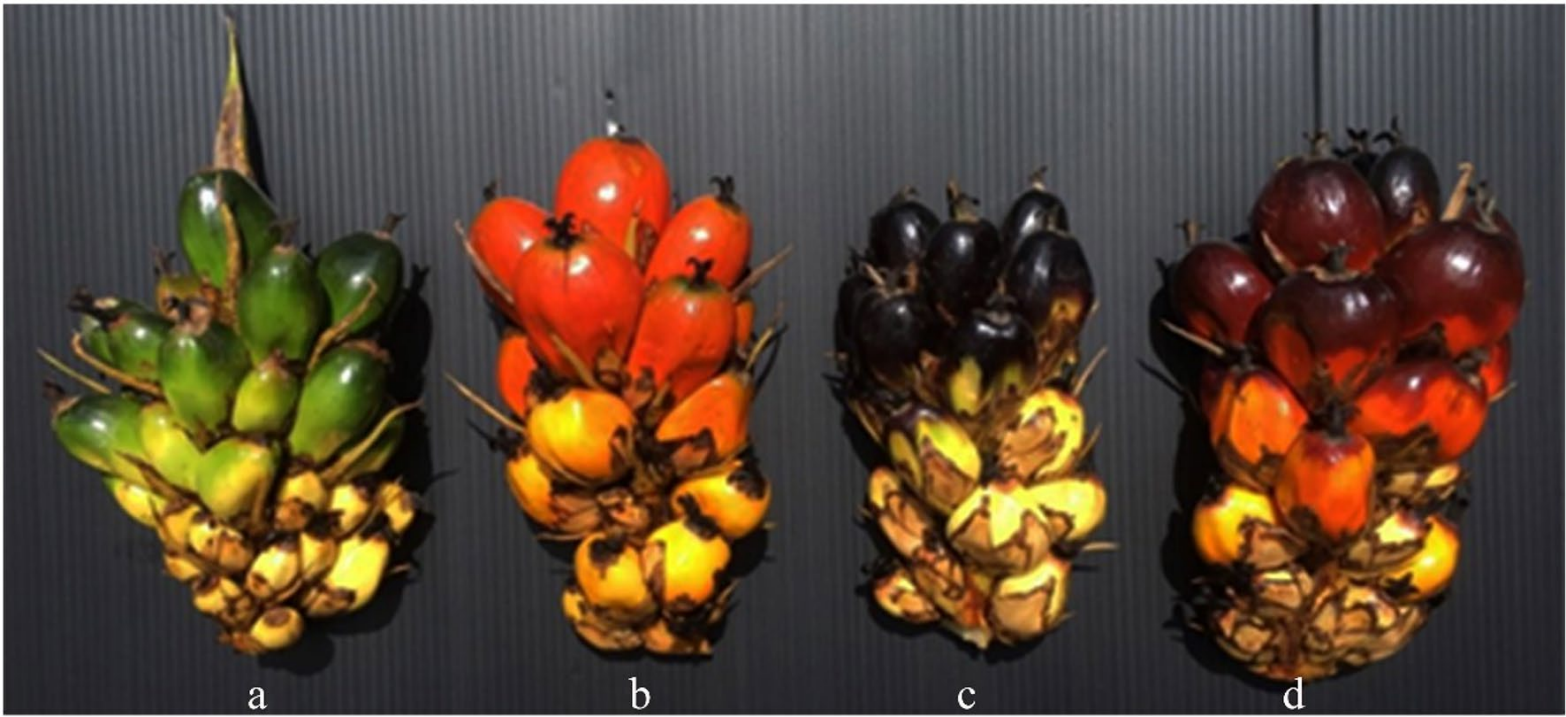
Exocarp morphotypes of oil palm fruit. (**a**) Virescent type; unripe. (**b**) Virescent type; ripe. (**c**) Nigrescent type; unripe. (**d**) Nigrescent type; ripe. (unripe: four weeks after fertilization, ripe: 15 weeks after fertilization).

Oil palm fruit of both the nigrescent type (Nigeria black) and virescent type (Deli × Calabar) at the unripe stage (four weeks after fertilization) and ripe stage (15 weeks after fertilization) (Fig. 7) were collected from 20-year-old oil palm trees growing at CPI Agrotech Co., Ltd., in Chumporn Province, Thailand. Three fruits of each morphotype were used as biological replicates. Total RNA from the exocarp of individual fruit was extracted using TriPure Isolation Reagent (Roche, Germany) following the manufacturer’s protocol. The integrity and purity of the total RNA were assessed on an Agilent Bioanalyzer 2100 (Agilent Technologies, CA, USA). Then, the RNA with the highest quality for each sample was selected and used for sequencing. Four cDNA libraries, unripe virescent (green exocarp; G_exo), ripe virescent (orange exocarp; Or_exo), unripe nigrescent (black exocarp; B_exo) and ripe nigrescent (red exocarp; R_exo), were prepared and sequenced by Novogene Bioinformatics Technology Co., Ltd. (Tianjin, China). Total RNA was treated with DNase I before eluting the mRNA with oligo (dT)-conjugated magnetic beads. The purified mRNA was then fragmented into small pieces before being reverse transcribed to their final cDNA library form in accordance with the protocol of a Illumina Stranded mRNA Prep kit (Illumina, Inc., USA).

### Sequencing, de novo assembly and annotation

The quality of the raw reads was analyzed by FASTQC (https://www.bioinformatics.babraham.ac.uk/-projects/fastqc/) before analyzing the data. Illumina Casava v1.8 software was used to eliminate reads whose bases had a Phred quality value score [Qphred = − 10log_10_(e)] below 30, reads containing adapter sequences and reads of low quality. After purity filtering was completed, the high-quality clean read data were de novo assembled using Trinity software^36^ with an optimized K-mer length of 25 and the default parameters for each morphotype to construct a unique consensus sequence. To eliminate redundant contigs in each morphotype, de novo assembly data were obtained from the hierarchical clustering Corset program^37^. Furthermore, contigs were clustered and assembled into transcripts using Cufflinks^38^.

The assembled unigene sequences were queried against public protein databases, such as the NR, Nt, SwissProt, and KOG databases, using BLASTX (v2.2.28) with an E-value threshold of ≤ 10^–5^. The Pfam database was searched using the HMMER v3.0 package. The KO database categories were analyzed against the KEGG Automatic Annotation Server (KAAS) (http://www.genome.jp/kegg/)^39^. GO annotations of the unigenes were obtained from the Blast2GO v2.5 program^40^ (http://www.geneontology.org).

### Identification and functional annotation of DEGs

To identify genes differentially expressed during fruit development, de novo transcriptome analysis was performed by the RSEM program. The mapped clean reads were converted into fragments per kilobase of transcript sequence per million base pairs sequenced (FPKM), resulting in total mapped reads and contigs capable of being normalized and then evaluated for gene expression. DEGs were first analyzed between stages and morphotypes and then analyzed between unripe/ripe virescent types (G_exo and Or_exo) and nigrescent types (B_exo and R_exo) using the DESeq R package (1.10.1)^41^. A |log_2_(ratio)|> 1 and adjusted *p* value < 0.05 were used as thresholds for determining the significance of the differential expression of the unigenes.

### EST-SSR primer development

Only the sequences of the DEGs upregulated in the virescent fruit type were searched for SSR loci using WebSat (http://wsmartins.net/websat/). The SSR motifs contained 10 di-, 6 tri-, 5 tetra-, 4 penta-, and 3 hexanucleotide repeats. The distance between two SSR sequences was set to ≥ 100 bp, with SSR repeat motif lengths of 2–6 bp. Primer3 Plus (http://www.bioinformatics.nl/cgi-bin/primer3plus/-primer3plus.cgi) was used for primers designed with the following main criteria: 50–60% CG content; 50–70 °C melting temperature; 50–65 °C annealing temperature; 18–20 bp optimum primer length; and 200–300 bp expected product size. The obtained primers were rechecked again with Oligo-Nucleotide Properties Calculator (http://biotools.nubic.northwestern.edu/Oligo-Calc.html). A total of five individuals, Nigeria black and Deli × Calabar, were used for analyses of polymorphisms. Whole-genomic DNA of each individual was extracted from fresh leaves using the modified cetyl-trimethylammonium bromide (CTAB) method^42^. The DNA concentration and integrity were measured with a NanoDrop ND2000 Spectrophotometer (Thermo Fisher Scientific, USA) and via 1% agarose gel electrophoresis. PCR was carried out in a total volume of 10 μL consisting of 1 μL of DNA (50 ng), 2 μL of 5 × HOT FirePol Blend Master Mix (Solis BioDyne, Estonia), 0.25 μL of each primer (10 μM), and 6.5 μL of nuclease-free water (Thermo Fisher Scientific, USA). The amplification conditions were as follows: 95 °C for 15 min; 35 cycles of 95 °C for 30 s, 50–65 °C for 30 s, and 72 °C for 1 min; and finally, 72 °C for 7 min before holding at 4 °C. The PCR product was then subjected to 1% agarose gel electrophoresis. For primer screening, the optimum annealing temperature was used for each sample, and the PCR product was subjected to 5% denaturing polyacrylamide gel electrophoresis. A clear band visualized by silver staining was assigned a value of 1, and a weak or no band at the same position was assigned a value of 0. The data were then analyzed for allele frequency.

## Supplementary Information

Supplementary Information.

## Data Availability

The datasets generated during the current study are available in the Oil palm fruit color repository, Accession No. CRA007679, https://bigd.big.ac.cn/gsa/browse/CRA007679.
