# Supplementary material for: Comparative transcriptome profiling and molecular marker development for oil palm fruit color
Source: Sci Rep. 2022 Sep 15;12:15507. doi: 10.1038/s41598-022-19890-2 (PMC9478095; doi:10.1038/s41598-022-19890-2)
Supplement: Supplementary file 1 — Supplementary Information. [file 41598_2022_19890_MOESM1_ESM.pdf]

**Comparative transcriptome profiling and molecular marker development for oil palm fruit color**

Potjamarn Suraninpong[a,b]* and Sunya Nuanlaong[a]

[a] *School of Agricultural Technology and food Industry, Walailak University, Nakhon Si Thammarat 80160, Thailand*

[b] *Biomass and Oil Palm Center of Excellence*

[*] *Corresponding author's e-mail: spotjama@mail.wu.ac.th*

**Supplement 1.** Statistical output from the sequencing of oil palm fruit with different exocarp colors.

| Sample | Total raw reads | Total clean reads | Clean bases (Gbp) | Error (%) | Q20 (%) | Q30 (%) | GC Content (%) |
|---|---|---|---|---|---|---|---|
| G_exo* | 66,162,228 | 64,521,874 | 9.7 | 0.01 | 97.77 | 94.25 | 50.16 |
| Or_exo | 48,017,160 | 46,896,912 | 7.0 | 0.01 | 97.47 | 93.76 | 48.61 |
| B_exo | 65,928,554 | 64,104,046 | 9.6 | 0.01 | 97.89 | 94.52 | 49.51 |
| R_exo | 53,237,442 | 51,932,430 | 7.8 | 0.01 | 97.67 | 94.13 | 49.31 |

*G_exo = green immature virescent type, Or_exo = orange mature virescent type, B_exo = black immature nigrescent type and R_exo = red mature nigrescent type

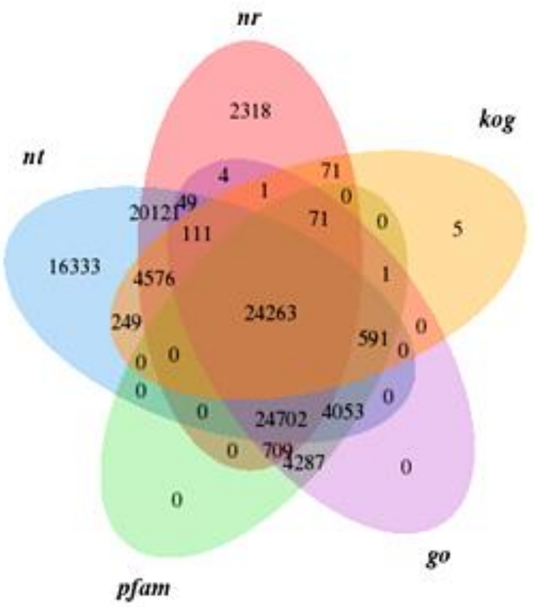


**Supplement 2.** Venn diagram displaying the unigene distribution according to data from five of the seven queried databases.

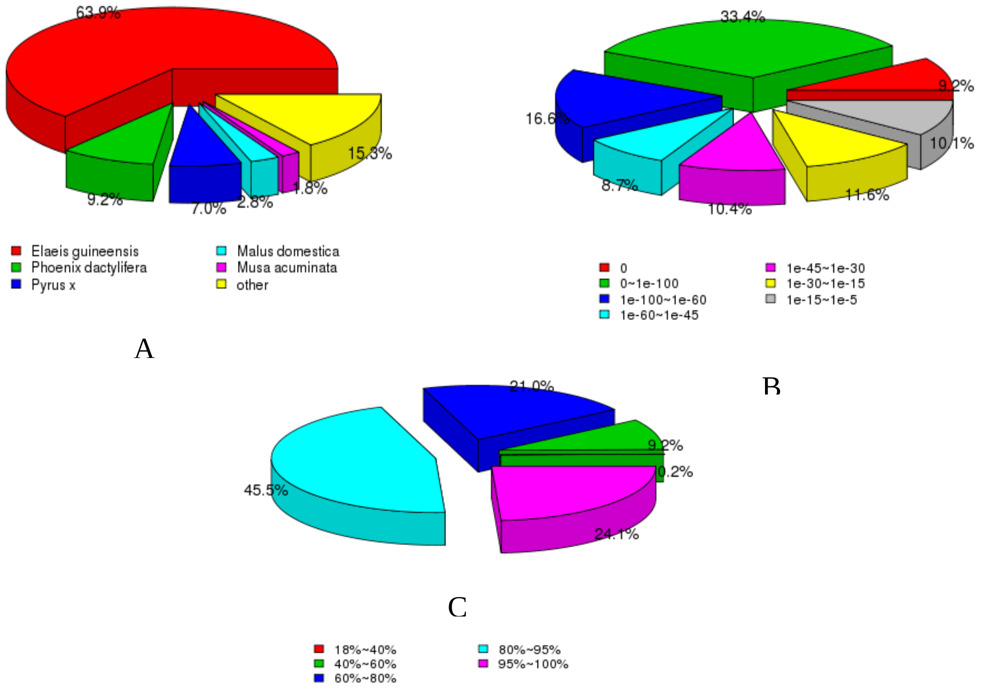


**Supplement 3.** Distribution of unigenes in the NR database. (**a**) Species distribution. (**b**) E-value distribution. (**c**) Similarity distribution.

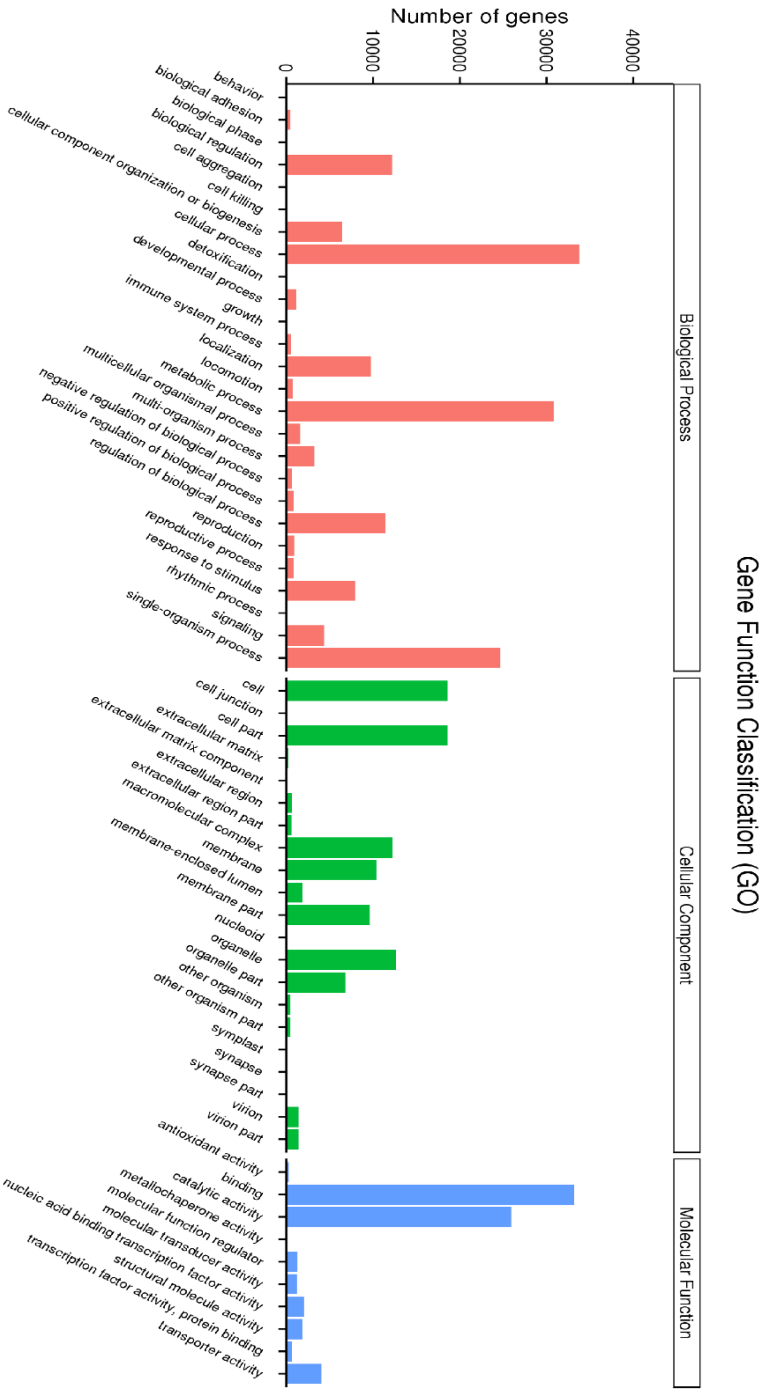


**Supplement 4.** GO annotations of specific homologous unigenes involved in oil palm fruit exocarp color.

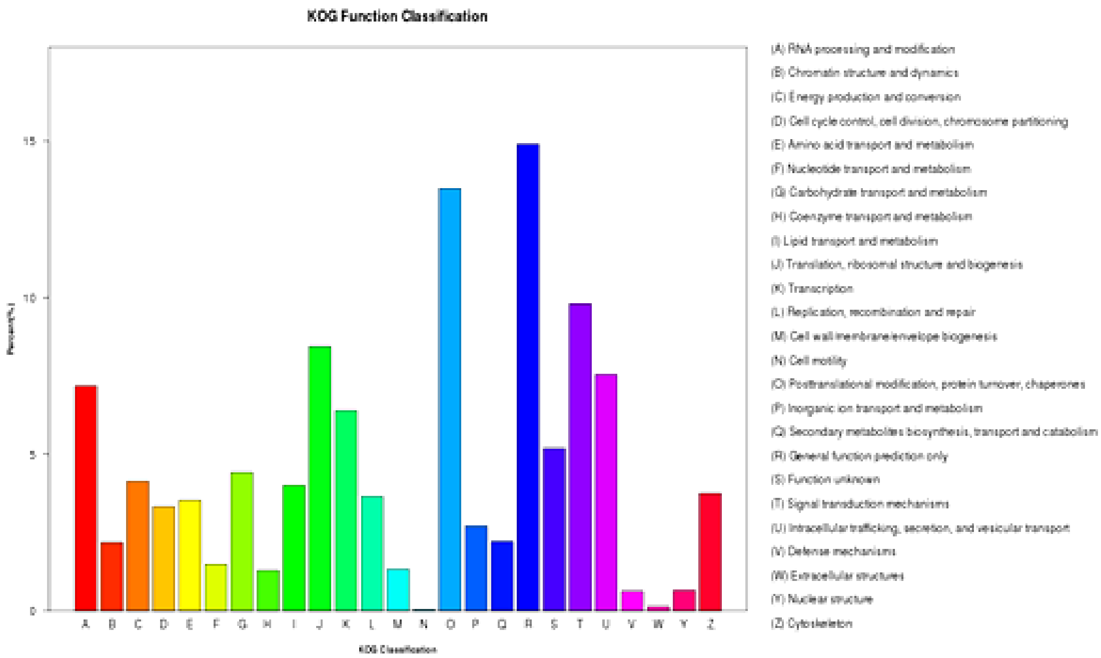


**Supplement 5.** KOG functional classification annotations of unigenes involved in oil palm fruit exocarp color.

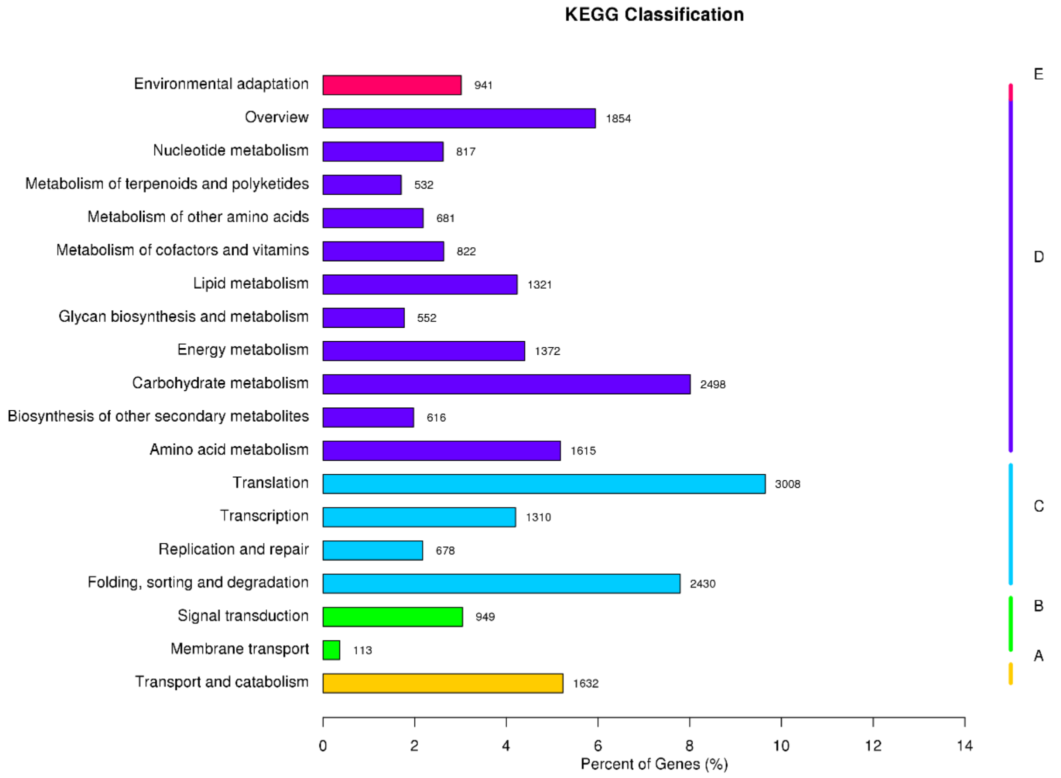


**Supplement 6.** KEGG pathway distribution of the unigenes involved in oil palm fruit exocarp color.

**Supplement 7.** Classification of transcription factors involved in oil palm fruit exocarp color.

| Transcription factor family | Number of unigenes |
|---|---|
| *C2H2* | 390 |
| *MYB* | 298 |
| *C3H* | 232 |
| *AP2-EREBP* | 225 |
| *Orphans* | 204 |
| *HB* | 201 |
| *bHLH* | 180 |
| *NAC* | 168 |
| *bZIP* | 167 |
| *SET* | 161 |
| *WRKY* | 154 |
| *SNF2* | 140 |
| *PHD* | 134 |
| *CCAAT* | 112 |
| *G2-like* | 111 |
| *FAR1* | 106 |
| *mTERF* | 98 |
| *GNAT* | 90 |
| *AUX/IAA* | 88 |
| *TRAF* | 85 |

**Supplement 8.** Postnormalization mapped reads of oil palm fruit exocarp color.

| Sample name | Clean read numbers | Total mapped reads |
|---|---|---|
| G_exo* | 64,521,874 | 51,741,008 (80.19%) |
| Or_exo | 46,896,912 | 35,436,652 (75.56%) |
| B_exo | 64,104,046 | 50,334,494 (78.52%) |
| R_exo | 51,932,430 | 39,778,974 (76.60%) |

* G_exo = green immature virescent type, Or_exo = orange mature virescent type, B_exo = black immature nigrescent type and R_exo = red mature nigrescent type

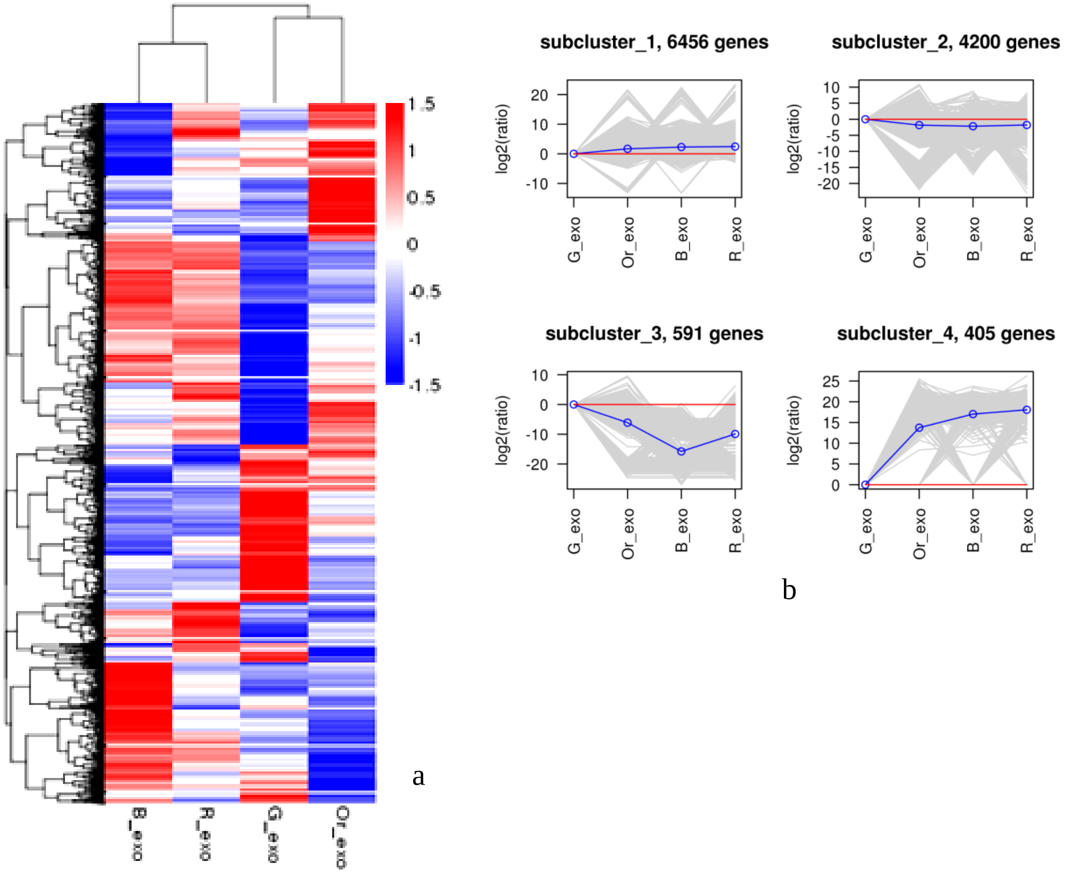


**Supplement 9.** RPKM analysis of oil palm fruit exocarp at different stages. (**a**) = Heatmap. (**b**) = K-means clustering. ( G_exo = green immature virescent type, Or_exo = orange mature virescent type, B_exo = black immature nigrescent type and R_exo = red mature nigrescent type)

**Supplement 10** Primers designed for DEGs involved in virescent-type oil palm fruit exocarp color.

| Gene | Repeated base | Forward primer (3’-> 5’) | Reverse primer (5’-> 3’) |
|---|---|---|---|
| 78405 | (TCC)$_{6}$ | CCA TGG GGG ATT TAA CTG | GAT TCC CAT CTA TCA TAC |
| 17088 | (GCACAT)$_{3}$ | CTG AAT GAC AGA GGA CAC TC | AAT ACA TCC CGC AAG CAG |
| 20186 | (AACCCT)$_{3}$ | CTC CCT TCC GTA GAG CTA TT | AGA CCA CTC GGA AAA AGC |
| 68477 | (GCC)$_{6}$ | TCT CCA GCG TCA GCT TC | GGC GAT GAT GAA GGC AAT GG |
| 60261 | (CAAAA)$_{4}$ | TTC ACC TGT AGA GTG CAC AG | ATG TGA AAG GCG TCC TCC AG |
| 26257.0 | (CGATCT)$_{3}$ | ATT CCT TGG TCT CGT CGT | GGT TCA CAC TCT TTC ACC TC |
| 54322 | (TGACGG)$_{5}$ | CCT CCT CCT AGT CGT TTC TT | GAA CAG GAA GGC GAT CTT |
| 16850 | (TCC)$_{6}$ | ACA CCA TGC TCC TTC CCT | AGA CGA TGG TGG AGT TGT C |
| 45258 | (GCG)$_{6}$ | AGT GGG GTG ACC TTC CTT | CTC ACC CCT CTG TAG TGC T |
| 55957 | (AG)$_{10}$ | CTC TAG ACG TTC TCC GAC AG | AGG AGG AGG GGA GAA GAG T |
| 69849-1 | (ACA)$_{7}$ | CTT AAC CTG TTC TGC TCC AG | GAG GCT CAT ACC TGA ATT GG |
| 69849-2 | (CCTTCC)$_{4}$ | GAG AAC TCT GGC TTT GTT GC | CCT GCA AGG GAT AGT ATC TG |
| 6292 | (GGAGG)$_{4}$ | GGG TGG AAA AAG AAG ACG | ATA ACT CTC CTC CCC ACG TC |
| 34708 | (CT)$_{9}$ | AGC GTG AGC TTG AAG CAG | TGG AGA GTG GGG GTT TAG |
| 9756 | (TATTTT)$_{3}$ | CTG GGA TCC AAA GGA GAC | GAT TCC CTC CCA TCA AAC |
| 13824 | (TCCACC)3 | CGA TGC TAG TCT TCA ACT GC | GAA GAC AGC AGC AAT AGC AC |
| 76163 | (ATT)6 | CTA TCA AAC TGG ATT CAG G | CCC CAG ATT GCA GAA GAT |
| 69369 | (AGGCCG)3 | GGG ATG ATA GTG AGG GAG TT | CCA CGT CTC CAT CAA ATC |
| 65133-1 | (GGA)6 | TCA CGC CAT ATA CCT CTC TC | GTG GTT TGA GAT CTC CTC AG |
| 65133-2 | (CAT)6 | CTT CTT CTG TAG CTG CCT GT | CAG AGT GCA TCA ACC TCA C |
| 59193 | (AAATAA)3 | ACT CTC AAG GGA GGC AAT | GGA CGA GAC GTA CCC TAA C |
| 30975-1 | (GGGGAA)3 | GGT AGG TCT TGA GGG CTT C | TTC ACA TCC ACT CCC TCC G |
| 30975-2 | (GAA)7 | CGG AGG GAG TGG ATG TGA A | AGT TTT CGA CGA CTC TCC GA |
| 67000 | (GCC)6 | TCT CCA GCG TCA GCT TC | GAA GGC AAT GGT GGA GGG |
| 45277 | (ATT)6 | GGT TCA TGC GAA AGC CCT TG | GGT GAT GGA GGA CAC TAC C |
| 42504 | (TTTTA)4 | GAC GAG GAG TGG GAG TTT AT | GAG ACG TCC TGC CTC TTA C |
| 46616 | (TCCGCC)4 | GGT CCT TGA AAT CGC TGT | GAG CAT GTA CCC CTT GAA GT |
| 8937 | (TGG)6 | TGG TGC AAA CGT CGA CGT | CAT GCC CTG AGA ATT AGG TC |
| 24168 | (TGG)6 | GGT GAG ATC CCC ATA GAA CT | GCA TAT GGA GCC ATC AGA |
| 9949 | (GCT)6 | CAC TTA TGA TAG TAG CCA GC | CTA AGT GCT ATC CAA ACA GC |
| 16480 | (AACAGC)3 | ATC CAT GGT GCT TGC TGG | CAG CTC TTT TTG GTC TTG C |
| 20987-1 | (GGAT)5 | CTG TCT TCT CAA CAA CCA CC | CCT TTC CGA CCA AAT TAC CC |
| 20987-2 | (AAAGAA)3 | GGG TAA TTT GGT CGG AAA GG | GTT TCG CGT TCA CTC TAC TC |
| 20718-1 | (AG)9 | TCG AGG CGG TGA AGA AAC | CCT TTC CCA GGA AGC AAT |
| 20718-2 | (GA)10 | CGA TTC CCT TCG GTG AAA | CAC AGC CTT GGA CGT GTT |
| 65806 | (CTCCCA)4 | CCG TGG TAT ATT GCG AGA | CTC CTC TAT CCA CCA CCA TA |
| 17659 | (CT)10 | GAC ATG ATC AAG TGC AGC TC | CCA TGA TCA CAA ACC GGC |
| 25568 | (AAAAT)4 | GCT TTG ACT GTC TGA TCT CG | GGC CCT CTT GTT AGC TAC TT |
| 68467-1 | (CGG)6 | CGC TGA GTG GCA AGA TTA | GAG AAC ATC AGC AGC TAA CC |
| 68467-2 | (CGG)7 | CTC AAA TCT CTT CCC GTC | AGA TGG TCC TAT TGA CAG GC |
| 16481 | (AACAGC)3 | GTC CTA CTC GAC TTC TTC CA | AGG AGG GGA AGA ACC AAA |
| 45460 | (CTG)13 | GAC AAG GAG GGA AGG AGA | GCC AAC CAG AGA AGG AAT |

**Supplement 10** Primers designed for DEGs involved in virescent-type oil palm fruit exocarp color (cont.).

| Gene | Repeated base | Forward primer (3’-> 5’) | Reverse primer (5’-> 3’) |
|---|---|---|---|
| 47524 | (AAATTT)3 | GTT CAT ATC ATG CCC CTG | GAA GAG GAG AGA ACG GAC A |
| 45307-1 | (CGC)6 | CTA CCG GTC CCA TAC TGA G | CTC AAT GAC AGT AGC CTC CA |
| 45307-2 | (CCGGCG)3 | AGG AGG CAT GTA CTG AAG C | AGA CGA ACA ACA CCA ACG |
| 29633 | (CGC)7 | CTT GGA CGT ACA CCT CGA T | AAG GAC AGG AGG TGG TGG T |
| 63980 | $(ACC)_7$ | GAG AAG AGG GTG AGT CTG GT | CTC TTG TTC CTC CTG CTG T |
| 50732 | $(GGGATT)_4$ | ACA CAC CGA GAT CTT CTC C | CCA TCC CCC TTA CAT TAC AC |
| 65628 | $(CT)_{10}$ | CTT CAG ACC CAT CTT CTC AC | GCT CTG ATA GCT GGT GAT TC |
| 48554 | $(AAAAT)_4$ | GCT CTG ATA GCT GGT GAT TC | GCA AGA ATA CCA GGC ACT |
| 45689 | $(TGG)_8$ | CTT CAC CTC CAG TTC CAT C | CTA AGG GGA AAT CCT CCA |
| 56840 | $(AGA)_6$ | GAT GCT AGC GAA ATC CTG | CGA TAT CGA CGT CTC CAA |
| 21057 | $(AT)_{11}$ | CTG ATT TCT ACG GCT TCT CG | CAG CTT ACC TCC TTT TCC AG |
| 28093 | $(GTG)_8$ | TGG CTT CCA TGG GTC CAT T | GAC AGC AAT CTC CTC AAC TC |
| 14470 | $(AAGA)_5$ | ATA GAA AAC GGG GAG GTA GG | CCT TTG GGA TAG AGG TGT C |
| 47731 | $(AAAAAG)_3$ | GAA CGA GGA AGG CAG TTT AG | TCC GGT TAG AAA CCC TTC |
| 47215 | $(TCTCT)_4$ | GCT CTC CAT CCT CTC TTC TC | CTG ACT CGC ATC ACA TCA C |
| 12019 | $(CCAAGC)_3$ | GGT GTG TTT CTC ATG GCT AC | ACT CCC GGT CAA GAA GAG |
| 59354 | $(AGG)_6$ | TCT ACC TGA GAG AGC GAG TC | GCT GTT CCT CCT CTT ATT CG |
| 45731-1 | $(CTCCT)_5$ | CGA GTA CGA GAA AGG ATG AG | AGC GAT GAT GGA AAA GAG |
| 45731-2 | $(TCG)_7$ | CTC TTT TCC ATC ATC GCT | GAC GTG AAC TCC AGA AAC |
| 4185.1 | $(TA)_9$ | CGT TGG ACT GTA CGA TCT C | GGC ACA AGT TAG GAA GGA TG |
| 40327 | $(TCTCCC)_3$ | TGC CTT TTC TTG GTC CTC | GCA GAA GCC ACA GAG ATA GT |
| 41883 | $(CTC)_8$ | GAG AGA CTG AGA TGG CTT CA | CCT TTT GAG CTC ACC ACT C |
| 9900 | $(AG)_9$ | GAA CCC TAA CCT GAT CCT TG | GCT TCA GAG ATG GCT ATG AG |
| 77543 | $(TATTTT)_3$ | CTG GGA TCC AAA GGA GAC | GAT TCC CTC CCA TCA AAC |
| 56652 | $(AAAAG)_4$ | GGA AAG TAA CAG GAC CCA GA | GAA GAC AAG CGA GGA GAA G |
| 15252 | $(TTAGCC)_3$ | CTG GTC TTC GTT CAA CTA CC | GAG ACG TGA GGA ACA AAG C |
| 55451 | $(CCGGCG)_3$ | CTC CTC CTC CCA GAA AAG | ACC GTG GAC TCC TTT GTT |
| 14314 | $(CTT)_6$ | ACT ACA TGA GCT GCT TGG TC | GGG ATT ACA TGT GCT ACG AC |
| 48901 | $(CCG)_6$ | TCA TCC ACC ACT GGT CCT | CGT CGT CGT AGT TCT TCC T |
| 8605-1 | $(TCTCCG)_3$ | GAC CGT GGA CAG GTA CAA | GAA GGG GAT GTC AGG TAA TC |
| 8605-2 | $(TCG)_6$ | GTG AGC GAT TAC CTG ACA TC | AGA GAT CAA TCG GTG GTG |
| 54491 | $(TTC)_6$ | CCT TCG CTC TGG TAT TGT | GAT CCA ATG GTT GAG GTC |
| 40074 | $(CT)_9$ | CCC TCC CCT CCT ATA AAG T | GTA AAG AAG ATG GGC CAG AC |
| 43587 | $(TCCTCG)_3$ | GGA AGC AGA GGA CGA TGT | GTG TGA CCG AGT CGG AGA AT |
| 64109-F | (TTC)6 | GGA AGG CGA ATG GGG TAA | CCT CCG TGT CAT TGA TGT G |
| 78725-F | $(AG)_9$ | AGA AGG CCC AGG AAA CTA C | ACC ACT TCG ATA CTC ACC AC |
| 18327-F | (CCGGTG)3 | TAC GCC GCC TTG TCC ACT A | TCT GGA GGT GGA GGA GGT CT |
| 7230-F | (TCG)6 | AAT CCC CAG CCA CAC GAA | ACG GTC TCA TCG AGG GTT T |
| 61220-F | (CGG)6 | CAG ATG ATC CCA GGA GCT A | GAG TTT GAG GGT CTC CAA G |
| 76494-F | (GAAGAG)3 | GCT TTT CTC TCC TCC AAC AG | GGC CAA GAT ACA GAG TGA GA |

**Supplement 11.** Allele and genotype frequency between nigrescent and virescent types of fruit of the Nigeria Black hybrid oil palm as revealed by different primers.

| Primer | Variety | Allele frequency | | | | Genotype frequency | | | | | | | | | |
|---|---|---|---|---|---|---|---|---|---|---|---|---|---|---|---|
| | | A | B | C | D | AA | AB | AC | AD | BB | BC | BD | CC | CD | DD |
| 20186 | Nir | 0.4 | 0.6 | . | . | . | 0.8 | . | . | 0.2 | . | . | . | . | . |
| | Vir | 0.6 | 0.4 | . | . | 0.2 | 0.8 | . | . | . | . | . | . | . | . |
| | all alleles | 1.0 | 1.0 | . | . | 0.2 | 1.6 | . | . | 0.2 | . | . | . | . | . |
| 54322 | Nir | 0.8 | 0.2 | . | . | 0.6 | 0.4 | . | . | . | . | . | . | . | . |
| | Vir | 0.4 | 0.6 | . | . | . | 0.8 | . | . | 0.2 | . | . | . | . | . |
| | all alleles | 1.2 | 0.8 | . | . | 0.6 | 1.2 | . | . | 0.2 | . | . | . | . | . |
| 55957 | Nir | 0.2 | 0.8 | . | . | . | 0.4 | . | . | 0.6 | . | . | . | . | . |
| | Vir | 0.1 | 0.9 | . | . | . | 0.2 | . | . | 0.8 | . | . | . | . | . |
| | all alleles | 0.3 | 1.7 | . | . | . | 0.6 | . | . | 1.4 | . | . | . | . | . |
| 76163 | Nir | 0.1 | 0.2 | 0.2 | 0.5 | . | . | . | 0.2 | . | . | 0.4 | . | 0.4 | . |
| | Vir | 0.1 | 0.2 | 0.2 | 0.5 | . | . | . | 0.2 | . | . | 0.4 | . | 0.4 | . |
| | all alleles | 0.2 | 0.4 | 0.4 | 1.0 | . | . | . | 0.4 | . | . | 0.8 | . | 0.8 | . |
| 9949 | Nir | . | 0.7 | 0.3 | . | . | . | . | . | 0.4 | 0.6 | . | . | . | . |
| | Vir | 0.1 | 0.9 | . | . | . | 0.2 | . | . | 0.8 | . | . | . | . | . |
| | all alleles | 0.1 | 1.6 | 0.3 | . | . | 0.2 | . | . | 1.2 | 0.6 | . | . | . | . |
| 8605-2 | Nir | 0.2 | 0.8 | . | . | . | 0.4 | . | . | 0.6 | . | . | . | . | . |
| | Vir | 0.1 | 0.9 | . | . | . | 0.2 | . | . | 0.8 | . | . | . | . | . |
| | all alleles | 0.3 | 1.7 | . | . | . | 0.6 | . | . | 1.4 | . | . | . | . | . |

(.= no allele or genotype)
